# Beneficial effect of TNF-α inhibition on diabetic peripheral neuropathy

**DOI:** 10.1186/1742-2094-10-69

**Published:** 2013-06-04

**Authors:** Xiaohong Shi, Yinghui Chen, Lubna Nadeem, Guoxiong Xu

**Affiliations:** 1 Department of Endocrinology, Jinshan Hospital, Fudan University, 1508 Longhang Road, Shanghai 201508, P.R China; 2 Department of Neurology, Jinshan Hospital, Fudan University, 1508 Longhang Road, Shanghai 201508, P.R China; 3 Department of Neurology, Shanghai Medical College, Fudan University, 138 Yixueyuan Road, Shanghai 200032, P.R China; 4 Research Centre for Women’s and Infants’ Health, Samuel Lunenfeld Research Institute, Mount Sinai Hospital, 600 University Avenue, Toronto, ON M5G 1X5, Canada; 5 Center Laboratory, Jinshan Hospital, Fudan University, 1508 Longhang Road, Shanghai 201508, P.R China

**Keywords:** Diabetes, Cytokine, TNF-α, rhTNFR:Fc, Nerve conduction velocity, Myelin basic protein

## Abstract

**Background:**

Tumor necrosis factor-α (TNF-α) is an important inflammatory factor produced by activated macrophages and monocytes and plays an important role in the pathogenesis of diabetic peripheral neuropathy (DPN). To evaluate the effect of TNF-α signaling suppression and the potential of TNF-α in the treatment of DPN, a recombinant human TNF-α receptor-antibody fusion protein (rhTNFR:Fc) was used. We focused on the pathophysiology of the sciatic nerve and examined the expression of myelin basic protein (MBP) under DPN status with or without TNF-α inhibition.

**Methods:**

The DPN rat model was generated by intraperitoneal injection of streptozotocin and by feeding with a high-fat, high-sugar diet. The nerve conduction velocity (NCV) in sciatic nerve of rat was monitored over a period of four weeks. The histopathological changes in nerve tissue were examined through traditional tissue histology and ultrastructure transmission electron microscopy (TEM). The expression of MBP was examined through western blot analysis.

**Results:**

The DPN induced rats showed significant signs of nerve damage including lower NCV, demyelination of nerve fibers, disorganization of lamellar and axonal structures, and decreased expression of MBP in the nerve tissue. The inhibition of TNF-α in the DPN rats resulted in a significant recovery from those symptoms compared to the DPN rats.

**Conclusions:**

Our study demonstrates that TNF-α plays a key role in the pathogenesis of DPN and its inhibition by rhTNFR:Fc can prove to be a useful therapeutic strategy for the treatment of and/or prevention from DPN symptoms.

## Background

Diabetic peripheral neuropathy (DPN) is a severe long-term complication of diabetes affecting about 50% diabetic patients [1,2]. It is a condition associated with progressive degeneration of nerve fibers [3] and has become a growing concern of researchers and clinicians due to increased prevalence and vague etiology. Over many years, the treatment of type 2 diabetes has been around the high blood sugar (glucose) toxicity. Even though blood sugar and blood pressure in many patients can be controlled, the development of DPN is neither preventable nor controlled. Studies have shown that the immune damage during the occurrence of type 2 diabetes may play a role in the development of DPN [4]. Recent evidences are being accumulated favoring the association between inflammation and type 2 diabetes, including its related complications like DPN [5,6]. Tumor necrosis factor-α (TNF-α) has been shown to play a central role in the pathogenesis of DPN [7]. Elevated levels of TNF-α and soluble TNF-α receptors (sTNFR1 and sTNFR2) have been reported in the serum of patients with DPN [8-10].

In this study, we used the previously described rat model of DPN [11], where DPN pathology was induced by streptozotocin administration, to evaluate the effect of TNF-α signaling suppression using recombinant human TNF-α receptor-antibody fusion protein. In order to investigate the beneficial effect of TNF-α inhibition on DPN, we focused on the pathophysiology of the sciatic nerve and measured the nerve conduction. The ultrastructure of the sciatic nerve and the expression of myelin basic protein (MBP) under DPN status with or without TNF-α inhibition were also examined.

## Materials and methods

### Reagents

Recombinant human tumor necrosis factor-α receptor II:IgG Fc fusion protein (rhTNFR:Fc) was obtained from Shanghai CP Guojian Pharmaceutical Co., Ltd (Shanghai, China). Rabbit anti-MBP monoclonal antibody was purchased from Sigma (St. Louis, MO, USA). HRP-conjugated goat anti-rabbit secondary antibody was purchased from Beijing Zhongshan Biotechnology Co., Ltd (Beijing, China).

### Experimental animals

Forty eight male Wistar rats (140 to 160 g weight, 6 weeks old) were purchased from Shanghai Animal Center, Medical College of Fudan University and housed in the animal facility for at least 5 days before use. All animal experiments were approved by the Ethic Committee of Animal Care of the Jinshan Hospital (# 2011-03) according to the Guidelines for Animal Experiments of the Chinese Academy of Medical Sciences. These animals were randomly divided into four groups (12 each): normal control, DPN, DNP plus low dose of rhTNFR:Fc (0.4 mg/kg, referred to as the DPN + T1), and DNP plus high dose of rhTNFR:Fc group (4 mg/kg, referred to as the DPN + T2). Animals were injected subcutaneously with rhTNFR:Fc in rhTNFR:Fc groups or with physiological buffered saline (PBS) as a vehicle in normal control and DPN groups twice per week for 4 weeks.

### Generation of diabetic peripheral neuropathy rats

The rat model of DPN was generated as previously described [11] with a slight modification. Briefly, the animals were fed with a high-fat, high-sugar diet (normal diet mixed with 10% lard and 20% sucrose) for 6 weeks. Diabetes was then induced by intraperitoneal injection of a single dose of streptozotocin (STZ, Sigma) at 30 mg/kg body weight in 0.1 M citrate buffer (pH 4.1). Diabetic animals were validated with the detection of high blood glucose level (>16.7 mmol/L), 48 hours after the STZ administration. Vehicle control animals were injected with the citrate buffer only. Following injection of STZ, the diabetic animals were continuously fed with a high-fat, high-sugar diet for another 4 weeks to generate DPN animals. The DPN model was validated by the lowering of nerve conduction velocity (NCV, <40 m/s). We monitored blood glucose levels during the experiment. One microliter of blood from the tail vein was used for measuring the level of blood glucose by the Fast Blood Glucose Monitoring System (Breeze 2, Bayer Healthcare, Mishawaka, IN, USA).

### Measurement of nerve conduction velocity

NCV was measured in the sciatic nerve. Briefly, rats were anesthetized by peritoneal injection with 10% chloral hydrate (0.3 ml/100 g) and fixed on a board. After disinfection of the proximal and distal ends of lower limb with alcohol, the electrodes were placed. For the determination of motor nerve conduction velocity (MNCV), the sciatic nerve was stimulated with single supramaximal square wave pulses (5 to 10 Ma and 40 μs duration) via fine needle electrodes inserted percutaneously in the sciatic notch and the ankle. The distance between the two sites of stimulation was 2 mm. MNCV was calculated by subtracting the distal latency from the proximal latency, and the result was divided into the distance between the stimulating and recording electrode. Sensory nerve conduction velocity (SNCV) was measured and recorded. The site of stimulation was located in the ankle and the recording site was in the sciatic notch. The maximal SNCV was calculated by measuring the latency to the onset/peak of the initial negative deflection and the distance between stimulating and recording electrodes, and the result was divided by latency period. All measurements were made in triplicate from each group.

### Preparation of sciatic nerve tissue and immunohistochemical staining

Rat was anesthetized by intraperitoneal injection of 10% chloral hydrate. The bilateral sciatic nerves were isolated and 1 cm of sciatic nerve tissue was fixed with 10% formaldehyde. The samples were then dehydrated and embedded in paraffin. After sectioning (5 μm thick) with a rotary slicer (LEICA RM2135, Wetzlar, Germany), hematoxylin and eosin stain (H&E) and luxol fast blue staining was performed to evaluate the neuronal damage and myelination status. The expression of MBP was examined by immunohistochemistry. Briefly, the section of sciatic nerve tissue was rinsed with 0.01 mol/L phosphate buffered saline (PBS, pH 7.2 to 7.4) three times, 5 min each, and then incubated with 10% normal rabbit serum at 37°C for 1 hour to block the non-specific binding. The sectioned tissue was then incubated with rabbit anti-rat MBP primary antibody (1:400 dilution, Sigma) at 37°C for 4 hours and subsequently at 4°C for 48 hours. After three times rinse with PBS, the sectioned tissue was incubated with horseradish peroxidase-conjugated goat anti-rabbit secondary antibody (1:2,000 dilution, Beijing Zhongshan Biotechnology Co., Ltd) at 37°C for 1 hour. After three times wash with PBS, the section was rinsed with 0.1 mol/L Tris–HCl buffer for 5 min. The section was then incubated in 0.05% DAB in 0.05 mol/L Tris–HCl buffer plus 3% hydrogen peroxide 1 to 2 drops for 5 to 15 min until the color was changed. The reaction was then stopped by dipping the slide into 0.05 mol/L Tris–HCl buffer. The slides were then dried and mounted with a cover slip. Rat brain tissue was used for positive control. For negative control, a normal rabbit serum (1:400 dilution) was used.

### Western blotting

Total protein was extracted from sciatic nerve tissue. Equal amount protein samples were subjected to SDS-PAGE and were transferred to PVDF membranes. After blocking with 1% skim milk in TBS-T at room temperature for 1 hour, the membranes were probed with rabbit anti-rat MBP or β-actin (1:500 dilution, Sigma) primary antibody at 4°C overnight and subsequently incubated with horseradish peroxidase-conjugated goat anti-rabbit secondary antibody (1:2,000 dilution) at room temperature for 2 hours. Signals were detected using ECL-Plus (Santa Clara, CA, USA) and quantified using Bio-Rad2000 gel imaging system with QUANTITY ONE software (Bio-Rad Laboratories, Hercules, CA, USA).

### Transmission electron microscopy

The sciatic never tissue was isolated as described before and placed in 2.5% glutaraldehyde. After the sample was cut into 1 × 1 × 3-4 mm, the nerve was divided into four parts longitudinally and fixed in 2.5% glutaraldehyde at 4°C overnight. The sciatic nerve tissue was post-fixated with 1% osmium tetroxide for 1 hour and then embedded in Epon812 resin. The tissue was cut into ultrathin sections (60 nm) and double stained with uranyl acetate and lead citrate for 10 min. After dehydration with ethanol and acetone, respectively, the samples were examined under transmission electron microscopy (Philips CM 120, Royal Dutch Philips Electronics Ltd, Eindhoven, Netherlands).

### Statistical analysis

All statistical analyses were carried out using SigmaStat (Chicago, IL, USA). Comparisons between groups were performed using either a paired student *t*-tests or one-way analysis of variance (ANOVA), where indicated. Data were presented as mean ± SEM. Differences were considered significant at values of *P* <0.05.

## Results

### Validation of diabetic peripheral neuropathy rat model

The DPN model was generated by high-fat, high-sugar diet for 6 weeks, followed by a single dose of STZ injection. After 48 hours of STZ administration, the DPN rats were validated by their higher blood glucose levels as compared with sham animals. We observed the higher glucose levels in DPN rat than normal control group (Table 1). However, there was no significant difference of the levels of blood glucose before (0 week) and after (4 weeks) the injection of rhTNFR:Fc in each group.

**Table 1:** Measurement of the levels of blood glucose

**Time**	**0 week**	**4 weeks**
CTL	6.26 ± 1.04	6.45 ± 0.92
DPN	19.58 ± 3.37^a^	20.78 ± 4.10
DPN + T1	20.95 ± 3.86^a^	20.58 ± 3.71
DPN + T2	19.95 ± 3.66^a^	20.53 ± 6.11

Note: Animals were injected subcutaneously with rhTNFR:Fc in rhTNFR:Fc-treated groups or with physiological buffered saline (PBS) as a vehicle in normal control (CTL) and in DPN groups twice per week for 4 weeks. Unit of blood glucose: mmol/L. DPN, diabetic peripheral neuropathy; T1, 0.4 mg/kg rhTNFR:Fc treatment; T2, 4 mg/kg rhTNFR:Fc treatment.

^a^Compared with group of CTL, *P* <0.05 (n = 12).

### Inhibition of TNF-α partially rescued the decrease of motor nerve conduction velocity and sensory nerve conduction velocity in diabetic peripheral neuropathy rat

**Figure 1:**
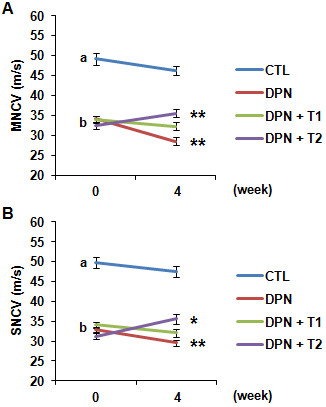
**Diabetic peripheral neuropathy (DPN)-induced change in motor nerve conduction velocity (MNCV) and sensory nerve conduction velocity (SNCV).** (**A**) Graph shows the rate of MNCV (m/s) in different groups at day zero and week 4 post-treatment of rhTNFR:Fc. DPN group showed significantly lower MNCV compared with the control group. High-dose rhTNFR:Fc group (DPN + T2) showed a significant recovery in MNCV compared with the DPN group. (**B**) Graph shows the rate of SNCV (m/s) in different groups at day zero and at week 4 post-treatment of rhTNFR:Fc. DPN group showed significantly lower SNCV compared with the control group. High-dose rhTNFR:Fc group (DPN + T2) showed a significant recovery in SNCV compared with the DPN group. All measurements were done in triplicate and data represent mean ± SEM (n = 12 per group). Statistical significance is denoted as: * *P* <0.05; ** *P* <0.01 (4 weeks versus 0 week); a (CTL) versus b (DNP), *P* <0.001.

The DPN status is characterized by the lowering of MNCV and SNCV. The MNCV and SNCV in different groups were measured before and after the treatment of rhTNFR:Fc. We found that animals with DPN had significantly lower MNCV and SNCV compared with control animals (Figure 1A and 1B; both *P* <0.001, a versus b), that was further decreased after four weeks. There was no statistical difference between the low-dose and high-dose groups, but the MNCV and SNCV values in the high-dose group (DPN-T2) were significantly higher than the DPN group of animals (Figure 1A, *P* <0.01 and 1B, *P* <0.05).

### TNF-α inhibition resulted in attenuation of the pathological changes of diabetic peripheral neuropathy

**Figure 2:**
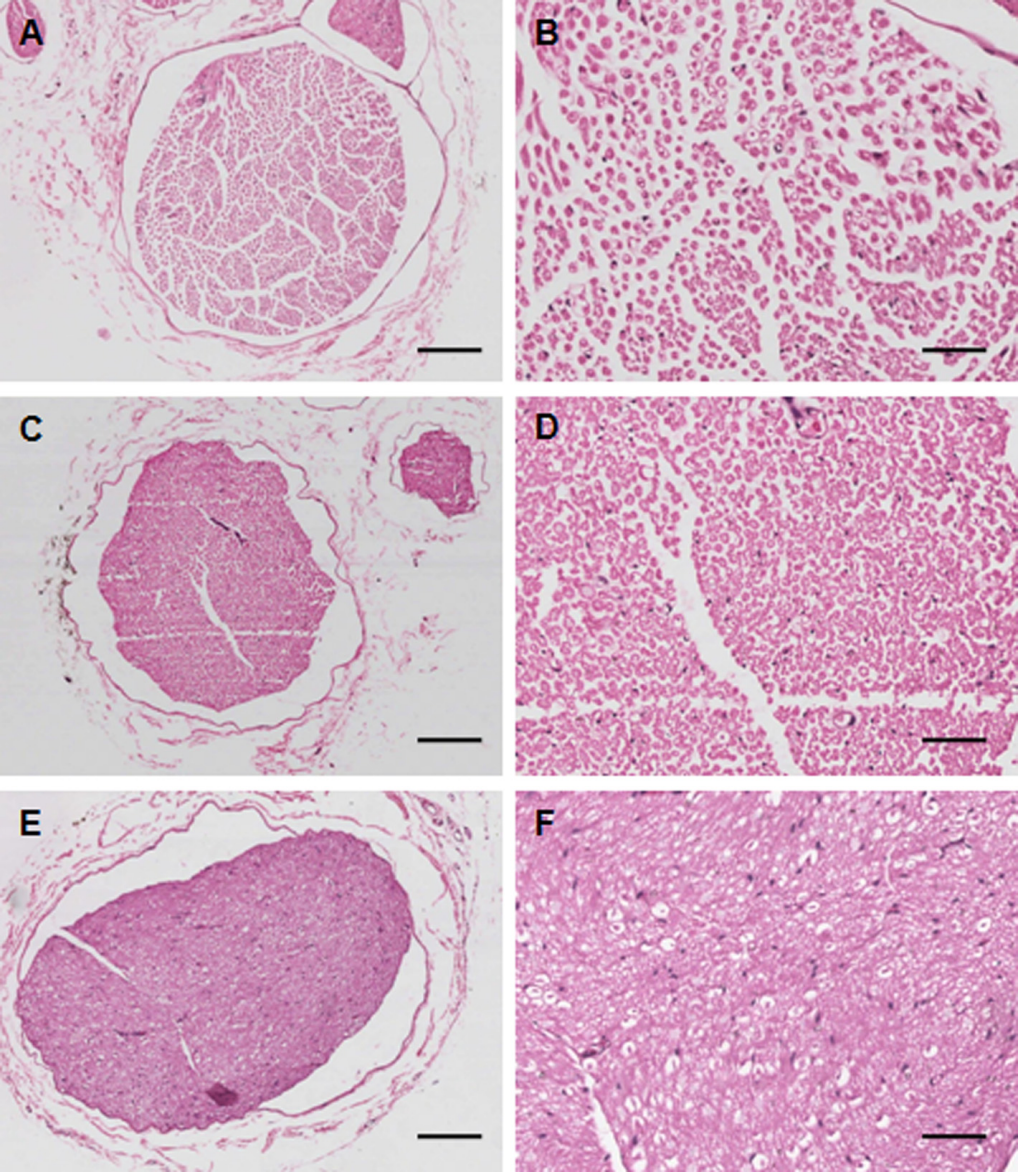
**Histological examination of hematoxylin and eosin (H&E) stained sciatic nerve.** (**A, B**): Normal control; (**C, D**): Diabetic peripheral neuropathy (DPN); and (**E, F**): High-dose rhTNFR:Fc (4 mg/kg) group (DPN + T2). Magnification: 100X (left panel, **A, C, E**), scale bars: 150 μm and 300X (right panel, **B, D, F**), scale bars: 50 μm.

To examine the histopathology, H&E staining of rat sciatic nerve was performed. In the control rats with normal glucose levels the myelinated nerve fibers were similar in size. Myelin appeared dense, round and uniform with ordered lamellar structure presenting neither axonal shrinkage nor its swelling. The wall of the endoneurial capillary was also even (Figure 2A and 2B). In DPN rats the myelin sheath of the myelinated nerve fibers was thin, loose, and disorganized and exhibited vacuolar-like defects (Figure 2C and 2D). Some nerve fibers in sciatic nerve appeared demyelinated. Lamellar spaces were expanded and separated from each other and visible signs of axonal atrophy were evident. The endoneurial capillary displayed thick wall and irregular lumen. The average cross-sectional area and the density of myelin nerve fibers was decreased in the DPN group as compared with the control group, while this decrease was partially restored in the DPN group treated with high-dose of rhTNFR:Fc (Figure 2E and 2F). The morphology of myelin in the TNF-α-inhibited DPN group was also improved compared with the DPN group and vacuolar-like degeneration was profoundly decreased.

**Figure 3:**
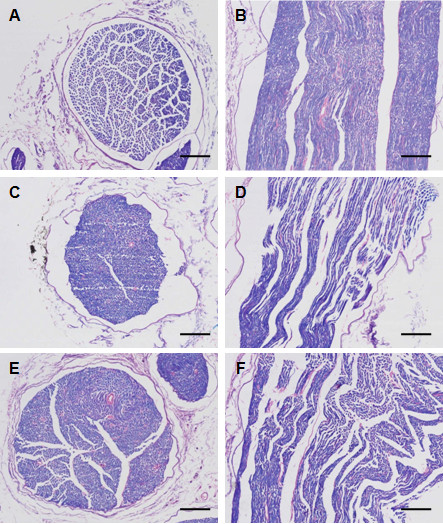
**Histological examination of luxol fast blue stained sciatic nerve.** (**A, B**): Normal control; (**C, D**): Diabetic peripheral neuropathy (DPN); and (**E, F**): High-dose rhTNFR:Fc (4 mg/kg) group (DPN + T2). Left panel shows cross-sections with 100X amplification (**A, C, E**, scale bar = 150 μm), and right panel shows longitudinal sections with 300X amplification (**B, D, F**, scale bar = 50 μm).

Next, we stained myelin with luxol fast blue dye in order to observe the changes and demyelination of the myelin sheath. This specific dye can bind to myelin imparting deep blue staining of myelin alone leaving a clear background with unstained axona and other structures. Under an optical microscope the cross-section of normal sciatic nerve showed central punctate appearance of myelin surrounding the nerve fibers (Figure 3A), while in longitudinal section myelin appeared as stacks of long, neat columns surrounded by basement membrane (Figure 3B). In the DPN rats the number of nerve fibers was reduced as compared with the control rats, depicted from both cross and longitudinal sections (Figure 3C and 3D). The diameter of nerve fibers in cross-section was markedly reduced suggesting the shrinkage of nerve fibers. Axon atrophy and myelin vacuolization was evident in the cross-sectional view of the sciatic nerve. Longitudinal sections depicted disruption, vacuolization, and shrinkage of myelin and swelling and atrophy of axons. An increase in Schwann cells was also observed. The blocking of TNF-α by rhTNFR:Fc showed marked improvement in the morphology of sciatic nerve in DPN + T2 group compared with the DPN group (Figure 3E and 3F). In cross section, the nerve fibers appeared as a circular, central punctate structure (Figure 3E).

**Figure 4:**
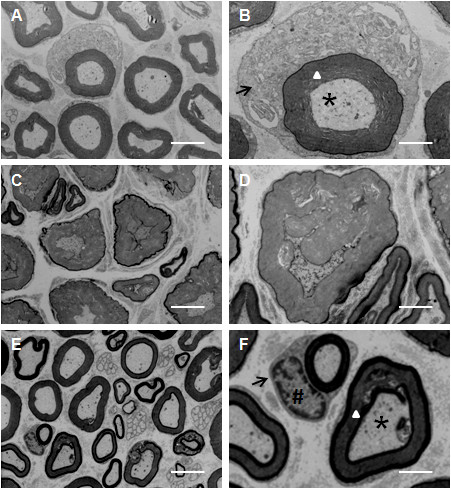
**Transmission electron microscopy of sciatic nerve.** (**A, B**): Normal control; (**C, D**): Diabetic peripheral neuropathy (DPN); and (**E, F**): High-dose rhTNFR:Fc (4 mg/kg) group (DPN + T2). Left panel (**A, C, E**) shows 4,000X magnification (Scale bars = 5 μm) and right panel (**B, D, F**) shows 13,000X magnification (Scale bars = 2 μm). Arrow indicates mitochondrion in Schwann’s cell, # key indicates the nucleus of Schwann’s cell, triangle indicates the myelin sheath, and the star indicates the axon.

Finally, we used transmission electron microscopy to examine the ultrastructure of myelin. The cross-section of sciatic nerve in normal rat presented uniform and dense myelination with structural integrity. Lamellar structures also showed concentric light and dark circles and axonal shrinkage and swelling (Figure 4A and 4B). In DPN rat model the myelin structure was disorganized and expanded toward both the axonal and the stromal sides (Figure 4C and 4D). There was a visible degree of lamellar fracture, acute demyelination, and separation of myelin sheath. Axonal microtubules and microfilaments were disrupted and disorganized demonstrating signs of atrophy. The ultra structure of Schwann cells showed irregular nuclei, expanded endoplasmic reticulum, vacuolization of mitochondria and ruptured basement membrane. The high-dose TNF-α-inhibited DPN rats (DPN + T2) showed dense and compact myelin structure and reduced vacuolar defects (Figure 4E and 4F). Although some lamellar separation was still there, the overall signs of pathology were mitigated.

### Inhibition of TNF-α reversed the decrease of myelin basic protein expression in the rats with the diabetic peripheral neuropathy

**Figure 5:**
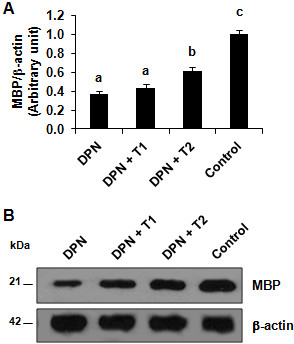
**Rescue of myelin basic protein (MBP) expression in diabetes peripheral neuropathy (DPN)-induced rats treated with rhTNFR:Fc.** Total protein was extracted and equal amount of protein from different samples was subjected to SDS-PAGE. Physiological buffered saline was used as a vehicle in normal control and DPN groups. DPN + T1, DNP plus low dose of rhTNFR:Fc (0.4 mg/kg); DPN + T2, DNP plus high dose of rhTNFR:Fc group (4 mg/kg). (**A**) Quantitative analysis, using densitometry of western blot from three independent experiments is shown. The expression of MBP was normalized to β-actin. Data represents mean ± SEM. Different letters denote significance, *P* <0.05, n = 12 experiments. (**B**) Representative western blot is shown.

Since MBP is critical for the myelination of nerve fibers, next we examined the protein expression of MBP in the sciatic nerve tissue. Compared with the control group, DNP group showed a significant decrease in the expression of MBP (Figure 5A and 5B). The low dose inhibition (0.4 mg/kg) of TNF-α in treatment group DPN + T1 caused a slight elevation of MBP expression, whereas the high dose inhibition (4 mg/kg) in treatment group DPN + T2 resulted in a significant increase of MBP expression compared to the DPN group (Figure 5A; *P* <0.05), reversing the DPN mediated decrease.

**Figure 6:**
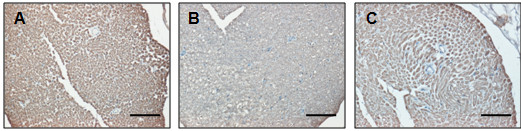
**Expression of myelin basic protein (MBP) by Immunohistochemistry.** Immunohistochemical staining of MBP in sciatic nerve. (**A**) Normal control; (**B**) Diabetic peripheral neuropathy (DPN); **C**) DPN + T2. Magnification = 300X, scale bars = 50 μm.

The MBP expression was also examined by the immunohistochemistry in the sciatic nerve. The control rats showed strong positive staining for MBP in the myelin sheath, whereas the capillaries and axons were MBP negative (Figure 6A). The expression of MBP in DPN group was lower as compared with the control group (Figure 6B), whereas it was higher in the DPN + T2 group (Figure 6C), indicating a protective effect of rhTNFR:Fc treatment over demyelination.

## Discussion

The damage of nerves (diabetic neuropathy) occurs due to high blood sugar levels from diabetes. About half of the people with diabetes develop nerve damage within 10 to 20 years post diagnosis. Diabetic Peripheral Neuropathy is one of the most common chronic complication of diabetes and the main reason for the disability occurring in type 2 diabetes. The mechanism of the development of DPN has not yet been fully elucidated. Previous studies have shown that it is a multifactorial disease and can occur due to genetic factors, hyperglycemia, abnormal fat or protein metabolism, and vascular abnormalities [12-14]. Several theories have been established to describe the etiology of DPN, including polyalcohol pathway, non-enzymatic protein glycosylation, abnormal lipid metabolism, oxygen-free radical damage, and the deficiency of neurotrophic factor [12,15]. Based on the pathogenesis, clinical treatments include the control of blood sugar, blood pressure, blood lipids, neurotrophic factors, antioxidants, and improvement of microcirculation. However, the relief from DPN symptoms is still not achieved. One study has shown that, despite intensive integrated intervention with an average follow-up of 13.3 years, there were about 55% of diabetic patients with DPN [16]. Therefore, it has been accepted that there must be some other factors simultaneously contributing to the pathology of DPN. Recent evidence has shown the involvement of immune factors in the occurrence of DPN [17,18]. Due to differences in genetic background, some patients may be more prone to autoimmune disorders. Under normal circumstances, the blood-nerve barrier functions to keep the circulating T cells in the immune tolerance state but long-term hyperglycemia can damage the vascular barrier of the nerves. Myelin protein glycosylation alters its antigenicity, rendering it vulnerable to the phagocytic attack of monocytes, macrophages, and neutrophils from blood circulation and tissue, and glial cells from nervous system that specifically recognize the glycosylated myelin antigen. In addition, the activated immune cells secrete cytokines such as TNF-α, IL-1β etcetera, which impart toxic effects on neurons and glial cells, leading to demyelination. The stimulated monocytes and endothelial cells have a vicious positive feed-back loop for secretion of inflammatory substances that potentiate nerve catastrophe.

Nerve biopsy in DPN patients has demonstrated that advanced glycation end products (AGEs) are mainly deposited in the axons and myelin sheath of nerve tissue [19]. It has been reported that AGEs can increase macrophage phagocytosis of nerve myelin. They execute this effect by elevating the expression of a variety of nuclear factors such as NF-kB-mediated inflammatory response genes, preventing NO-dependent vasodilatation and anticoagulation, resulting in segmental demyelination of the nerve cells [19].

Previous studies have also shown that the expression of TNF-α in the blood of DPN patients and rat DPN model is elevated. TNF-α is an important immune cytokine involved in the developmental process of many inflammatory, infectious, and autoimmune diseases and functions to kill or inhibit tumor cells, increase the phagocytic activity of neutrophils, and stimulate the production of other cytokines [9]. The pathological changes of DPN are characterized by axonal degeneration in unmyelinated fibers, while demyelination and diffused shrinkage in myelin nerve fibers [20,21]. The immune response, in which self-antigen component gets exposed, is the main reason leading to demyelination in case of DPN. T-cells activated by these antigens produce various cytokines, including TNF-α, which has a potential positive feed-back cycle to further raise its own immune response to mediate the inflammatory reaction [9]. It has been found that TNF-α expression leads to the oligodendrocyte toxicity and demyelination. It stimulates monocytes and endothelial cells to secrete IL-1β and IL-6 and other inflammatory factors to amplify or enhance its effect indirectly [22]. Furthermore, TNF-α inhibits the nitric oxide synthase (NOS) activity in vascular endothelial cell, resulting in a decrease of NO-induced vasodilatation. It has been shown to promote the expression of several growth factors and cell adhesion molecules, resulting in endothelial dysfunction and pathogenesis including stenosis, hemodynamic abnormalities, decreased perfusion, and neurotrophic blood vessels damage [23,24].

To further confirm the role of TNF-α in the DPN pathogenesis, in the present study, we applied subcutaneous injection of a TNF-α receptor-antibody fusion protein (rhTNFR:Fc) to the DPN-induced rats in order to inhibit TNF-α. The results showed that rhTNFR:Fc treatment in rats with DPN significantly increased NCV and MNCV values, particularly in high-dose group indicating that rhTNFR:Fc effectively delayed the progress of the DPN in rats with DPN. This was further confirmed by histological and TEM ultrastructure examination of the sciatic nerve. The sciatic nerve of DPN-induced rats showed the typical pathological features including thinning and destruction of the myelin sheath and disorganization of the lamellar structures. Inhibition of TNF-α with rhTNFR:Fc, however, resulted in a marked improvement of sciatic nerve and its component’s morphology. This beneficial effect of targeting TNF-α is in agreement with a recent study that pharmacological inhibition of TNF-α by etanercept, a TNF-α antagonist, blocks behavioral signs of diabetic neuropathic pain [25].

Furthermore, we found an increase in MBP expression was observed in the rhTNFR:Fc treated groups, implicating that TNF-α is the main cause of the demyelination of the sciatic nerve. MBP is a strong basic membrane protein produced in the oligodendrocyte cells of vertebrate central nervous system and Schwann cells of peripheral nervous system. It is the major constituent protein of the nervous system myelin and is mainly localized to the myelin serosa. BMP binds to the myelin lipids to maintain the myelin structure and functional stability and promotes the process of myelination. When the nerves are damaged, especially in the incidence of demyelination on the nerve, MBP expression is reported to decrease in the nerves with a subsequent increase in serum MBP levels. Currently, serum MBP is used as an indicator of the degree of CNS and myelin damage. rhTNFR:Fc has been applied in clinical practice in the treatment of rheumatoid arthritis, psoriasis, and ankylosing spondylitis. It is also reported to be useful in the treatment of various inflammatory diseases in rat animal models [26]. Our data supports the notion that rhTNFR:Fc may prove to be useful for the treatment of DPN patients.

## Conclusions

Thus, rhTNFR:Fc, a recombinant human type II tumor necrosis factor receptor-antibody fusion protein, acts as a competitor of TNF-α in the blood for binding to the TNF-α receptor on the cell surface, thereby blocking its activity. This study confirms that the immune cytokine TNF-α is directly involved in the pathogenesis of DPN, and inhibiting its activity can improve DPN symptoms in terms of electrophysiological and morphological changes. Our findings show that TNF-α is one of the key mediators of the immune inflammatory response that induces DNP and implies that rhTNFR:Fc is a powerful candidate that should be considered for the treatment or prevention therapy of DPN. The specific mechanism of immune damage on DPN, however, still requires further study to develop reliable therapeutic strategies and alleviate the potential of DPN disorder particularly in patients suffering from diabetes.

## Abbreviations

AGEs: Advanced glycation end products; DPN: Diabetic peripheral neuropathy; H&E: Hematoxylin and eosin; MBP: Myelin basic protein; MNCV: Motor nerve conduction velocity; NCV: Nerve conduction velocity; rhTNFR:Fc: recombinant human TNF-α receptor-antibody fusion protein; SNCV: Sensory nerve conduction velocity; STZ: Streptozotocin; TEM: Transmission electron microscopy; TNF-α: Tumor necrosis factor-α.

## Competing interests

The authors declare that they have no competing interests.

## Authors’ contributions

XS and YC designed study and performed the experiments. YC and GX carried out the statistical analysis. YC, LN and GX analyzed the data. YC and LN drafted manuscript. GX finalized figures and wrote the final version of the manuscript. All authors read and approved the final manuscript.

## References

[B1] Wooten K Clinical features and electrodiagnosis of diabetic peripheral neuropathy in the dysvascular patient Phys Med Rehabil Clin N Am 2009 20 657 676 10.1016/j.pmr.2009.06.011 19781504

[B2] Tesfaye S Selvarajah D Advances in the epidemiology, pathogenesis and management of diabetic peripheral neuropathy Diabetes Metab Res Rev 2012 28 Suppl 1 8 14 22271716 10.1002/dmrr.2239

[B3] Boulton AJ Vinik AI Arezzo JC Bril V Feldman EL Freeman R Malik RA Maser RE Sosenko JM Ziegler D Diabetic neuropathies: a statement by the American Diabetes Association Diabetes Care 2005 28 956 962 10.2337/diacare.28.4.956 15793206

[B4] Krendel DA Costigan DA Hopkins LC Successful treatment of neuropathies in patients with diabetes mellitus Arch Neurol 1995 52 1053 1061 10.1001/archneur.1995.00540350039015 7487556

[B5] Wang Y Schmeichel AM Iida H Schmelzer JD Low PA Enhanced inflammatory response via activation of NF-kappaB in acute experimental diabetic neuropathy subjected to ischemia-reperfusion injury J Neurol Sci 2006 247 47 52 10.1016/j.jns.2006.03.011 16631800

[B6] Schram MT Chaturvedi N Schalkwijk CG Fuller JH Stehouwer CD Markers of inflammation are cross-sectionally associated with microvascular complications and cardiovascular disease in type 1 diabetes–the EURODIAB Prospective Complications Study Diabetologia 2005 48 370 378 10.1007/s00125-004-1628-8 15692810

[B7] Satoh J Yagihashi S Toyota T The possible role of tumor necrosis factor-alpha in diabetic polyneuropathy Exp Diabesity Res 2003 4 65 71 10.1155/EDR.2003.65 14630568 PMC2478597

[B8] Zoppini G Faccini G Muggeo M Zenari L Falezza G Targher G Elevated plasma levels of soluble receptors of TNF-alpha and their association with smoking and microvascular complications in young adults with type 1 diabetes J Clin Endocrinol Metabol 2001 86 3805 3808 10.1210/jc.86.8.3805 11502815

[B9] Gonzalez-Clemente JM Mauricio D Richart C Broch M Caixas A Megia A Gimenez-Palop O Simon I Martinez-Riquelme A Gimenez-Perez G Vendrell J Diabetic neuropathy is associated with activation of the TNF-alpha system in subjects with type 1 diabetes mellitus Clin Endocrinol (Oxf) 2005 63 525 529 10.1111/j.1365-2265.2005.02376.x 16268804

[B10] Moriwaki Y Yamamoto T Shibutani Y Aoki E Tsutsumi Z Takahashi S Okamura H Koga M Fukuchi M Hada T Elevated levels of interleukin-18 and tumor necrosis factor-[alpha ] in serum of patients with type 2 diabetes mellitus: Relationship with diabetic nephropathy Metab Clin Exp 2003 52 605 608 10.1053/meta.2003.50096 12759891

[B11] Reed MJ Meszaros K Entes LJ Claypool MD Pinkett JG Gadbois TM Reaven GM A new rat model of type 2 diabetes: the fat-fed, streptozotocin-treated rat Metabolism 2000 49 1390 1394 10.1053/meta.2000.17721 11092499

[B12] Harati Y Diabetic neuropathies: unanswered questions Neurol Clin 2007 25 303 317 10.1016/j.ncl.2007.01.002 17324729

[B13] Tesfaye S Chaturvedi N Eaton SE Ward JD Manes C Ionescu-Tirgoviste C Witte DR Fuller JH Vascular risk factors and diabetic neuropathy N Engl J Med 2005 352 341 350 10.1056/NEJMoa032782 15673800

[B14] Tavakkoly-Bazzaz J Amoli MM Pravica V Chandrasecaran R Boulton AJ Larijani B Hutchinson IV VEGF gene polymorphism association with diabetic neuropathy Mol Biol Rep 2010 37 3625 3630 10.1007/s11033-010-0013-6 20352346

[B15] Miranda-Massari JR Gonzalez MJ Jimenez FJ Allende-Vigo MZ Duconge J Metabolic correction in the management of diabetic peripheral neuropathy: improving clinical results beyond symptom control Curr Clin Pharmacol 2011 6 260 273 10.2174/157488411798375967 22082324 PMC3682498

[B16] Gaede P Lund-Andersen H Parving HH Pedersen O Effect of a multifactorial intervention on mortality in type 2 diabetes N Engl J Med 2008 358 580 591 10.1056/NEJMoa0706245 18256393

[B17] Said G Lacroix C Lozeron P Ropert A Plante V Adams D Inflammatory vasculopathy in multifocal diabetic neuropathy Brain 2003 126 376 385 10.1093/brain/awg029 12538404

[B18] Gundogdu BM Diabetic peripheral neuropathy: an update on pathogenesis and management Curr Neurol Neurosci Rep 2006 6 1 4 16469264 10.1007/s11910-996-0001-3

[B19] Lappas M Permezel M Rice GE Advanced glycation endproducts mediate pro-inflammatory actions in human gestational tissues via nuclear factor-kappaB and extracellular signal-regulated kinase 1/2 J Endocrinol 2007 193 269 277 10.1677/JOE-06-0081 17470518

[B20] Boulton AJ Malik RA Arezzo JC Sosenko JM Diabetic somatic neuropathies Diabetes Care 2004 27 1458 1486 10.2337/diacare.27.6.1458 15161806

[B21] Sinnreich M Taylor BV Dyck PJ Diabetic neuropathies. Classification, clinical features, and pathophysiological basis Neurologist 2005 11 63 79 10.1097/01.nrl.0000156314.24508.ed 15733329

[B22] Fromont A De Seze J Fleury MC Maillefert JF Moreau T Inflammatory demyelinating events following treatment with anti-tumor necrosis factor Cytokine 2009 45 55 57 10.1016/j.cyto.2008.11.002 19109035

[B23] Malik RA Newrick PG Sharma AK Jennings A Ah-See AK Mayhew TM Jakubowski J Boulton AJ Ward JD Microangiopathy in human diabetic neuropathy: relationship between capillary abnormalities and the severity of neuropathy Diabetologia 1989 32 92 102 10.1007/BF00505180 2721843

[B24] Estrella JS Nelson RN Sturges BK Vernau KM Williams DC LeCouteur RA Shelton GD Mizisin AP Endoneurial microvascular pathology in feline diabetic neuropathy Microvasc Res 2008 75 403 410 10.1016/j.mvr.2007.12.002 18207200 PMC2413429

[B25] Dogrul A Gul H Yesilyurt O Ulas UH Yildiz O Systemic and spinal administration of etanercept, a tumor necrosis factor alpha inhibitor, blocks tactile allodynia in diabetic mice Acta Diabetol 2011 48 135 142 10.1007/s00592-010-0237-x 21104419

[B26] Zhang C Cai S Chen P Chen JB Wu J Wu SJ Zhou R Inhibition of tumor necrosis factor-alpha reduces alveolar septal cell apoptosis in passive smoking rats Chin Med J (Engl) 2008 121 597 601 18466678

